# Modified Appleby procedure for pancreatic body cancer: Retrograde gastric and hepatic perfusion via a replaced left hepatic artery – A vascular paradigm shift: A case report

**DOI:** 10.1016/j.ijscr.2025.111865

**Published:** 2025-08-24

**Authors:** Supreet Kumar, Rigved Gupta, Sandeep Vohra, Vivek Tandon, Deepak Govil

**Affiliations:** a Department of Surgical Gastroenterology and GI Oncology, Indraprastha Apollo Hospitals, New Delhi, India; b Department of Hepato-Pancreato-Biliary Surgery, Indraprastha Apollo Hospitals, New Delhi, India; c Department of Radiodiagnosis, Indraprastha Apollo Hospitals, New Delhi, India; d Department of Surgical Gastroenterology and GI Oncology, Indraprastha Apollo Hospitals, New Delhi, India

**Keywords:** Modified Appleby procedure, Pancreatic body cancer, Replaced left hepatic artery, Retrograde gastric perfusion, Celiac axis resection, Vascular anatomical variant, Case report

## Abstract

**Introduction and importance:**

Pancreatic body cancers encasing the celiac axis present a formidable surgical challenge, historically deemed inoperable due to the risk of gastric and hepatic ischemia. The modified Appleby procedure, involving distal pancreatectomy with en bloc celiac axis resection, offers curative potential in carefully selected cases. We report a **rare and uncharted vascular variant** that enabled safe execution of this procedure with preservation of organ perfusion.

**Case presentation:**

A 58-year-old male presented with epigastric discomfort, weight loss, and a 4-cm pancreatic body tumor encasing the celiac axis. Contrast-enhanced CT and 3D angiography revealed a **replaced left hepatic artery (LHA) originating from the left gastric artery (LGA)**, with strong collateral flow from the superior mesenteric artery (SMA). During surgery, the gastroepiploic arcade was sacrificed. However, intraoperative Doppler confirmed a novel collateral circuit—**SMA → GDA → PHA → segment 4 → segments 2/3 → LGA via replaced LHA—preserving both hepatic and gastric perfusion.** The procedure was completed without complications. Postoperative recovery was uneventful apart from transient delayed gastric emptying. Histology confirmed a moderately differentiated pancreatic ductal adenocarcinoma with R0 resection.

**Clinical discussion:**

This is the first reported case demonstrating effective retrograde gastric perfusion via a replaced LHA following celiac axis resection. It highlights how variant anatomy, if preserved, can be safely leveraged to extend resectability criteria in pancreatic cancer.

**Conclusion:**

Meticulous vascular mapping and intraoperative perfusion assessment can unlock curative surgical options in anatomically complex pancreatic tumors.

## Highlights

• Modified Appleby procedure successfully performed for pancreatic body tumor encasing the celiac axis.
• Unique retrograde gastric perfusion preserved via a replaced left hepatic artery arising from the left gastric artery.
• Novel collateral route: SMA → GDA → PHA → segment 4 → segments 2/3 → LGA via replaced LHA.
• Intraoperative Doppler confirmed arterial pulsatility despite gastroepiploic arcade sacrifice.
• Case demonstrates how anatomical variants can redefine vascular strategy and expand surgical resectability.

## 1 Introduction

Pancreatic ductal adenocarcinoma (PDAC) remains one of the most aggressive solid malignancies globally, with a 5-year survival rate that rarely exceeds 10 % despite significant advances in systemic therapies and surgical strategies [1,2]. Tumors located in the body and tail of the pancreas account for approximately 30–35 % of all pancreatic cancers, and are often diagnosed late due to their deep retroperitoneal location and nonspecific symptomatology [3]. This delay frequently results in vascular encasement—particularly of the celiac axis—rendering such tumors historically unresectable [4].

The advent and evolution of the **Modified Appleby Procedure**—distal pancreatectomy with en bloc resection of the celiac axis—has redefined the boundaries of resectability in pancreatic body cancers [5]. First adapted from gastric cancer surgery, this technique enables R0 resection in carefully selected patients by relying on retrograde hepatic perfusion from the superior mesenteric artery (SMA) via the pancreaticoduodenal arcades and gastroduodenal artery (GDA) [6]. When combined with meticulous preoperative planning and real-time intraoperative perfusion assessment, it has shown promising oncologic outcomes without compromising visceral viability [7,8].

However, a major challenge arises in preserving **gastric perfusion**, especially in scenarios where the gastroepiploic arcade is sacrificed due to tumor proximity. While retrograde hepatic flow is well-studied, maintenance of gastric viability in such cases is less frequently addressed in literature, making it a crucial yet underappreciated element of the procedure [9].

Arterial anatomical variations—particularly those involving the hepatic vasculature—occur in nearly 20 % of individuals and can substantially influence surgical strategy [10]. A **replaced left hepatic artery (LHA)** arising from the **left gastric artery (LGA)** is a known but relatively rare variant, occurring in approximately 3–7 % of cases [11]. Though often considered an anatomical curiosity, such variants may hold significant clinical value during extended resections where traditional vascular pathways are compromised.

In this context, we report a **first-in-literature case** where a replaced LHA enabled **retrograde gastric perfusion via a collateral network involving the SMA, GDA, PHA, segment 4, and segment 2/3 branches**, ultimately supplying the LGA and preserving gastric viability. This novel vascular route challenges conventional paradigms and underscores the importance of integrating detailed preoperative 3D vascular mapping with intraoperative Doppler flow assessment.

Our case highlights how awareness and intelligent utilization of arterial variants can safely expand resectability boundaries in pancreatic body cancers, ensuring not only oncological clearance but also preservation of organ perfusion in anatomically demanding scenarios. This case report has been reported in line with the SCARE checklist [24].

## 2 Case presentation

A 58-year-old man with no significant past medical history presented with a three-month history of intermittent epigastric discomfort, early satiety, and unintentional weight loss of approximately 5 kg. There were no associated gastrointestinal symptoms such as jaundice, vomiting, or gastrointestinal bleeding. Physical examination was unremarkable except for mild epigastric tenderness. Laboratory workup revealed elevated serum CA 19–9 at 294 U/mL, with normal liver enzymes and bilirubin.

**Fig. 1:**
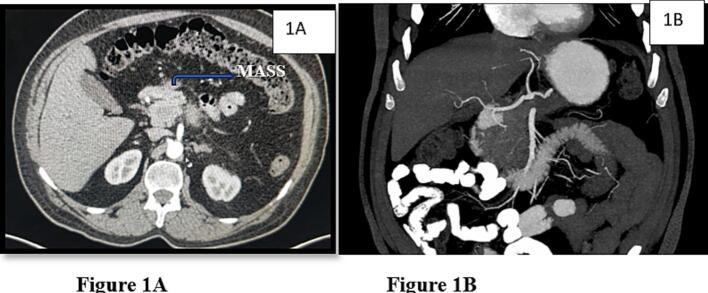
A. Axial contrast-enhanced CT showing a 4-cm hypodense mass in the body of the pancreas encasing the celiac axis. The lesion demonstrates loss of fat planes and circumferential contact with the celiac trunk, indicating locally advanced pancreatic ductal adenocarcinoma. B. Coronal CT image highlighting the tumor's relationship to the celiac axis and proximal splenic artery. The image emphasizes the loss of intervening soft tissue and the tumor's proximity to key vascular landmarks.

Cross-sectional imaging with contrast-enhanced computed tomography (CT) revealed a 4-cm ill-defined, hypodense mass in the body of the pancreas. The tumor encased the celiac axis circumferentially and abutted the splenic artery and vein (Fig. 1A, B). There was no evidence of hepatic or distant metastases, lymphadenopathy, or ascites.

**Fig. 2:**
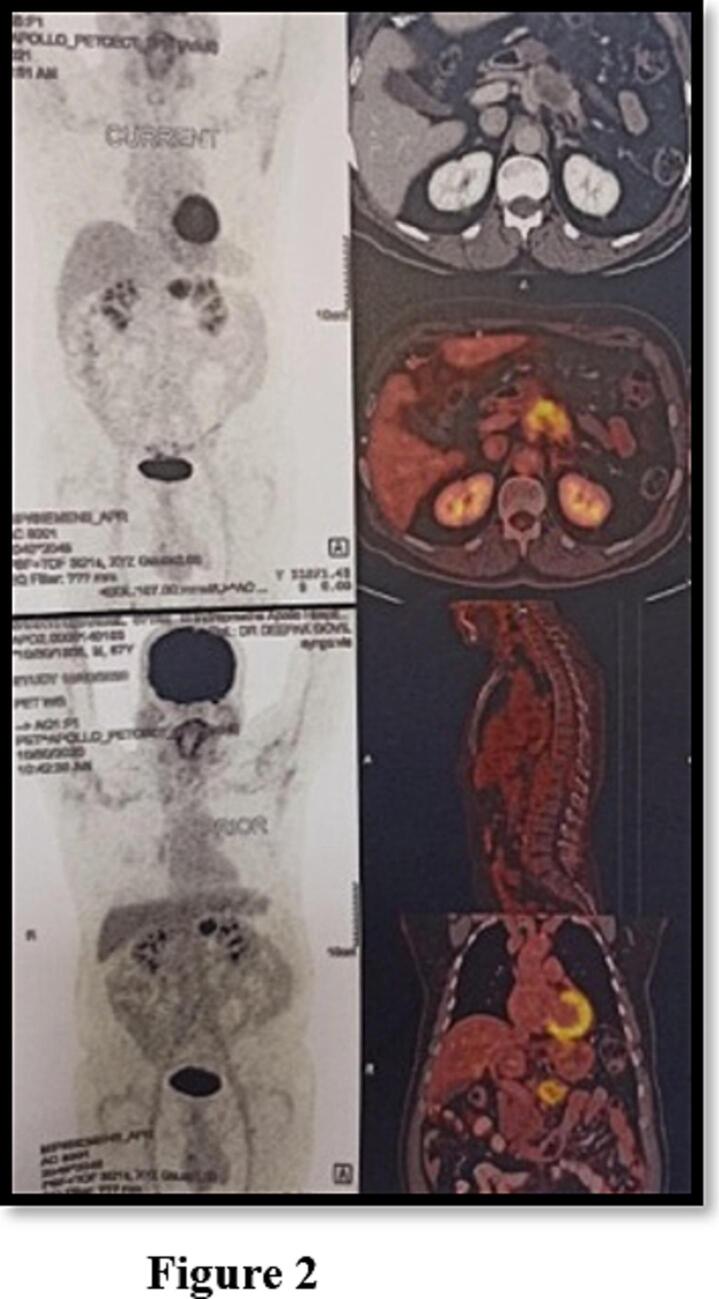
PET-CT scan demonstrating intense FDG uptake in the pancreatic body tumor with no evidence of distant metastases. The hypermetabolic signal correlates with the mass seen on cross-sectional imaging and supports surgical exploration in a non-metastatic setting.

Positron emission tomography–CT (PET-CT) demonstrated a metabolically active lesion restricted to the pancreatic body, with no systemic spread (Fig. 2). Endoscopic ultrasound confirmed the mass encasing the celiac axis and proximal splenic vessels, and fine-needle aspiration revealed moderately differentiated pancreatic ductal adenocarcinoma (PDAC) with a Ki-67 index of 15 %. Immunohistochemistry was negative for neuroendocrine markers.

**Fig. 3:**
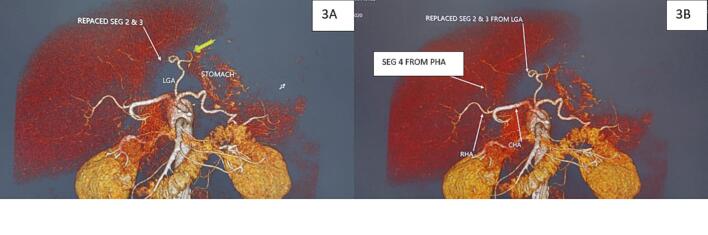
A. Three-dimensional CT angiography revealing a replaced left hepatic artery (LHA) arising from the left gastric artery (LGA). Prominent collateral connections from the superior mesenteric artery (SMA) via the gastroduodenal artery (GDA) to the proper hepatic artery (PHA) are visualized, indicating a variant anatomy conducive to collateral perfusion. B. Schematic illustration of the novel retrograde collateral pathway: SMA → GDA → PHA → segment 4 branches → segments 2/3 → LGA via replaced LHA. This pathway played a critical role in preserving hepatic and gastric perfusion post–celiac axis resection.

Three-dimensional CT angiography and multiplanar reconstruction revealed a rare arterial anomaly: **a replaced left hepatic artery (LHA) arising from the left gastric artery (LGA).** Notably, there was robust collateral communication between the superior mesenteric artery (SMA) and the proper hepatic artery (PHA) via the gastroduodenal artery (GDA), with further intrahepatic connections between segment 4 and the segment 2/3 arteries. These vessels appeared to form a vascular bridge supplying the LGA retrogradely through the replaced LHA (Fig. 3A, B).

In view of excellent performance status, absence of nodal or distant disease, and a strong patient preference to avoid delay, the multidisciplinary tumor board (MDT) approved upfront surgery. The rationale for foregoing neoadjuvant therapy was based on favourable tumor biology, lack of vascular invasion beyond celiac encasement, and anticipated technical feasibility of achieving R0 resection, hence the patient was deemed a candidate for modified Appleby procedure (distal pancreatectomy with en bloc celiac axis resection). He was optimized preoperatively with nutritional support, incentive spirometry, and enrolled in an Enhanced Recovery After Surgery (ERAS) protocol.

### 2.1 Operative details

**Fig. 4:**
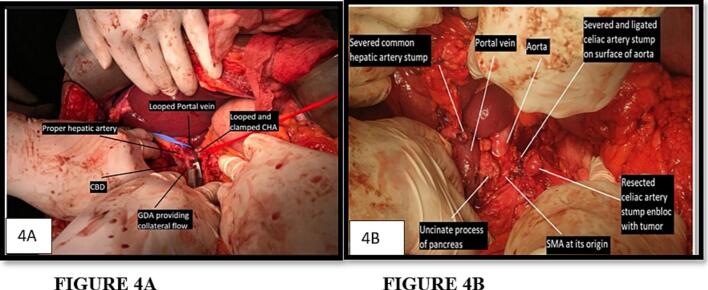
A. Surgical image demonstrating meticulous dissection of the vascular structures with the looping of Portal vein, Common Hepatic artery, Gastroduodenal artery. Also denoted in the image is the clamping of the common hepatic artery with confirmation of blood flow through the Gastroduodenal artery. B. Intraoperative image showing meticulous dissection and mobilization of the distal pancreas and spleen along the anterior RAMPS (Radical Antegrade Modular Pancreatosplenectomy) plane, achieving oncologic clearance.

Following diagnostic laparoscopy that excluded peritoneal or hepatic deposits, a midline laparotomy was performed. The spleen and distal pancreas were mobilized along the anterior RAMPS (Radical Antegrade Modular Pancreatosplenectomy) plane (Fig. 4A).

The tumor was found to encase the celiac axis and proximal splenic artery. Due to fibrosis and proximity, the gastroepiploic arcade was sacrificed. The left gastric artery (LGA) did arise from the celiac trunk, and its origin was indeed in close proximity to the zone of encasement. However, intraoperative dissection revealed that the tumor encased the celiac trunk distal to the LGA take-off, allowing preservation of the LGA without compromising oncologic clearance. This decision was reinforced by intraoperative frozen section analysis of the pancreatic neck margin, which confirmed an R0 resection. This helped the surgical team meticulously preserve the segment 2 and 3 gastric branches arising via the replaced LHA.

Fig. 9 illustrates the proposed schematic of retrograde arterial perfusion following celiac axis resection, demonstrating how the preserved LGA and replaced LHA facilitated continued gastric and hepatic viability.

**Fig. 5:**
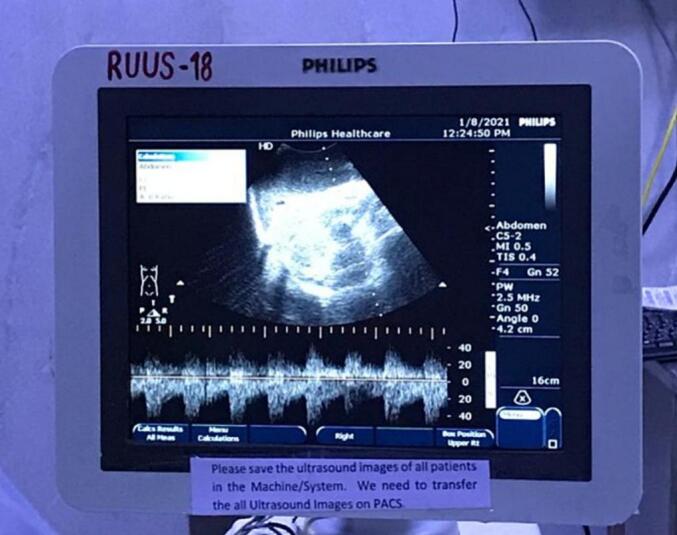
Intraoperative Doppler ultrasound assessment following temporary clamping of the common hepatic artery (CHA) confirms retrograde pulsatile flow in the proper hepatic artery (PHA), validating SMA-mediated collateral circulation and enabling safe progression with arterial resection.

Prior to proceeding with arterial transection, a test clamp of the celiac trunk was performed. **A 12-minute ischemia window was observed**, during which Doppler ultrasonography was used to assess perfusion which despite CHA occlusion, demonstrated that the PHA exhibited robust retrograde pulsation, confirming collateral flow from the SMA via the GDA and pancreaticoduodenal arcades. Doppler parameters showed a **peak systolic velocity (PSV) of 44 cm/s** and a **resistive index (RI) of 0.65** in the PHA, and confirmed triphasic waveforms in the segment 4 and 2/3 branches and the gastric arcade vessels (Fig. 5). Palpation and colour Doppler confirmed gastric viability.

**Fig. 6:**
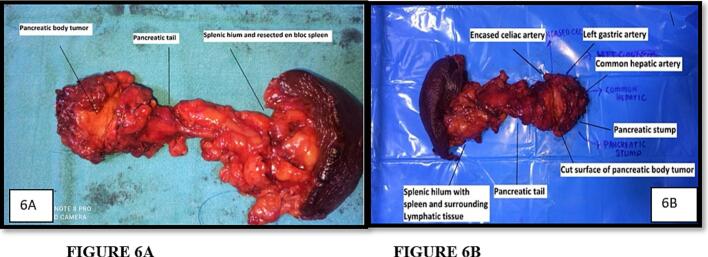
A. Distal Pancreatectomy with en bloc Celiac Axis resection with splenectomy Final Specimen. B. Post-resection surgical specimen showing pancreatic body (with tumor) and tail of pancreas with severed vascular stumps of Celiac artery, Common Hepatic artery and Left Gastric artery, Spleen with perihilar lymphatic tissue.

An en-bloc distal pancreatectomy, splenectomy, and celiac axis resection were performed uneventfully (Figs. 4B, 6A, B). Intraoperative frozen section was performed at the pancreatic neck transection margin and confirmed negative margins (R0 resection).

Blood loss was 320 mL, operative duration was 270 min, and no transfusion was required. The preserved vascular anatomy was further visualized post-dissection, confirming uninterrupted arterial inflow to the stomach and liver. A triple lumen Freka's tube was placed which as both an access for feeding and gastric decompression.

### 2.2 Postoperative course

The patient was extubated on-table and transferred to the high-dependency unit for intensive perfusion monitoring. Enteral nutrition was initiated on postoperative day 2 via nasojejunal tube, with transition to oral feeds on day 4.

He developed transient delayed gastric emptying (DGE), classified as ISGPS **Grade B** requiring prokinetics and continued NJ feeding until day 6, after which gastric motility normalized. No ischemic gastric or hepatic complications were observed.

**Fig. 7:**
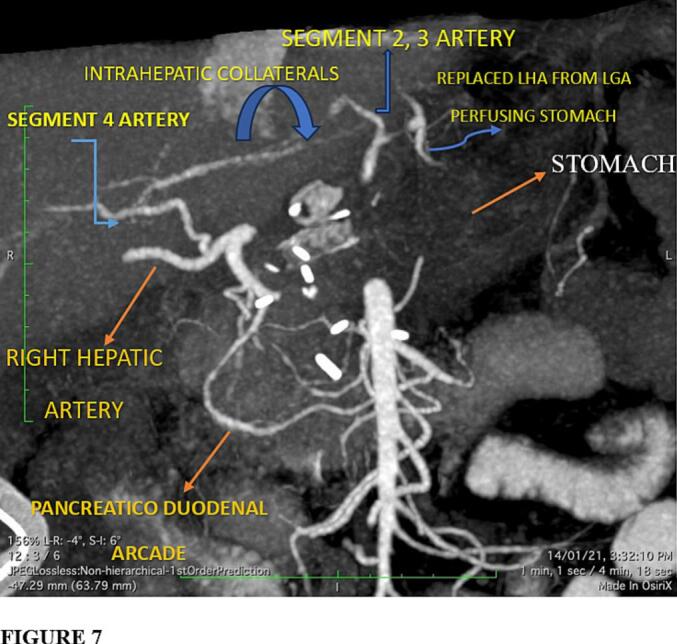
Postoperative contrast-enhanced CT on day 7 demonstrating preserved gastric and hepatic arterial perfusion. No ischemic changes were identified, validating the intraoperative perfusion assessment.

**Fig. 8:**
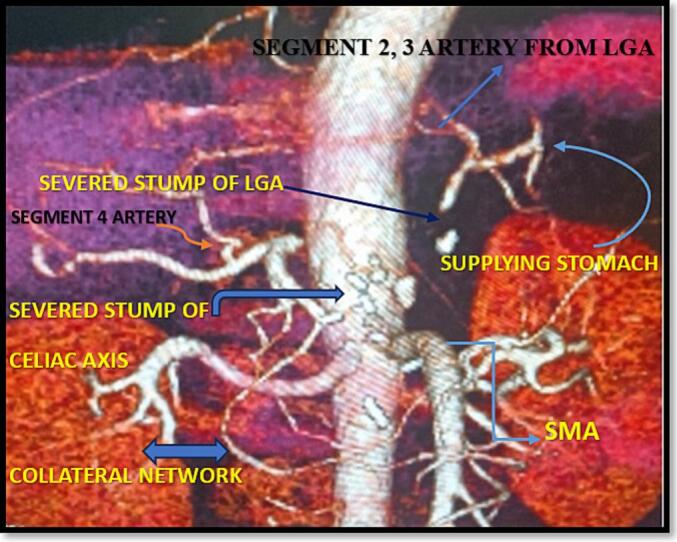
CT angiogram showing intact collateral flow to the liver and stomach postoperatively. 3D Reconstructed images confirm that the SMA-derived pathway (from the SMA through GDA, PHA, and segmental intrahepatic vessels toward the LGA and gastric territories) successfully perfused the stomach via the described retrograde route, ensuring dual-organ viability after modified Appleby procedure.

A **Grade A** biochemical pancreatic fistula was noted, with drain amylase on day 3 exceeding serum levels but without clinical sequelae. It resolved spontaneously. CT angiography and Doppler on day 7 confirmed preserved hepatic and gastric perfusion, affirming the intraoperative findings (Fig. 7, Fig. 8).

The patient was discharged in stable condition on postoperative day 10.

Final histopathology revealed a moderately differentiated PDAC with an R0 resection margin and lymphovascular invasion in 2 out of 18 lymph nodes (pT2N1M0, AJCC 8th edition). He was referred for adjuvant modified FOLFIRINOX chemotherapy. He is subsequently under the follow-up of Medical Oncologist.

## 3 Discussion

Pancreatic body and tail adenocarcinomas that encase the celiac axis traditionally signalled inoperability due to the high risk of ischemic complications following arterial resection. However, the advent of modified Appleby procedure (MAP)—distal pancreatectomy with en bloc celiac axis resection (DP-CAR)—has expanded the boundaries of curative surgery in select patients [[12], [13], [14]]. Key to this advancement is the capacity to preserve hepatic and gastric perfusion through alternate arterial routes.

The **retrograde hepatic perfusion pathway**—originating from the SMA and traversing the inferior pancreaticoduodenal artery, pancreatic arcades, GDA, and into the PHA—is well recognized and forms the cornerstone of the MAP's feasibility [15]. Yet, gastric perfusion, often jeopardized by the sacrifice of the right gastroepiploic and short gastric arteries, remains a less discussed but equally critical concern. Inadequate gastric inflow can result in ischemia, delayed gastric emptying (DGE), and even necrosis adversely affecting postoperative recovery and nutrition [16,17].

In the present case, the presence of a replaced LHA arising from the LGA and its integration into a previously undescribed retrograde collateral route ensured uninterrupted perfusion of both the liver and stomach, despite celiac axis resection and loss of the gastroepiploic arcade. The **pathway traced was: SMA → GDA → PHA → Segment 4 branches → Segment 2/3 arteries → replaced LHA and finally the LGA** —a novel vascular circuit not previously documented. This route, verified intraoperatively via Doppler ultrasonography. Although direct Doppler interrogation of the replaced LHA was technically limited, robust flow was observed in downstream branches, including the left hepatic ductal arcade and segment IV and confirmed postoperatively by CT angiography, offers a paradigm shift in how arterial variants can redefine resectability in locally advanced PDAC.

**Fig. 9:**
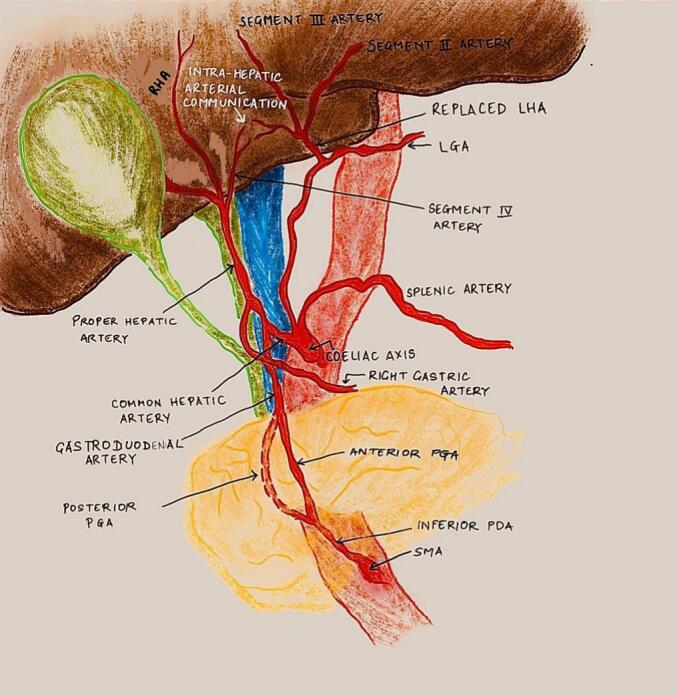
Schematic representation of the modified arterial perfusion circuit following celiac axis resection in a patient with a replaced left hepatic artery (LHA) arising from the left gastric artery (LGA). The pancreas and surrounding structures are colour-coded for anatomical clarity. This schematic highlights the critical role of functional collateral pathways in maintaining hepatic and gastric viability after extended pancreatectomy.

This variant-based advantage holds surgical significance beyond curiosity. The replaced LHA is reported in ∼3–7 % of individuals [18], often dismissed as an incidental finding. However, when the LGA is preserved, and retrograde channels are viable, such a configuration becomes a lifeline for gastric perfusion, as demonstrated here. Awareness of this possibility should inform surgical planning and dissection strategy—prioritizing LGA preservation even when celiac resection is necessary. A schematic diagram (Fig. 9) has now been added to illustrate the proposed retrograde perfusion pathway: arterial inflow from the aortic origin of LGA → replaced LHA → segment IV branches → communicating arcades with segments II/III and gastric vascular beds. This hypothesized circuit may represent an under-recognized collateral route and warrants further angiographic exploration.

**Anatomical classifications** such as those of Michels and Hiatt categorize arterial variants by origin but do not account for **functional retrograde potential**, which is arguably more relevant in extended resections. We propose the need for future anatomical-taxonomical frameworks that integrate **flow directionality and collateral connectivity**, offering surgeons a more functional understanding of variant implications during complex HPB procedures.

Preoperative 3D CT angiography and multiplanar reconstruction were instrumental in identifying this vascular anatomy. However, the future of preoperative planning may lie in AI-based perfusion prediction models, integrating flow dynamics, vessel diameter, resistance indices, and anatomical variants to stratify perfusion risk. Emerging technologies such as virtual surgical simulation and automated perfusion mapping could further personalize vascular strategy in MAP candidates [19].

Intraoperatively, **real-time Doppler ultrasonography** served as the critical gatekeeper to safe progression. The detection of retrograde pulsatility in the PHA and viable waveforms in the segmental gastric arteries confirmed physiological integrity before irreversible arterial resection. **ICG fluorescence angiography** is another tool employed in similar scenarios [20]. The absence of ICG fluorescence imaging is a limitation of this report. However, the combination of Doppler-based flow confirmation and postoperative clinical markers (stable liver enzymes, early enteral tolerance, and no signs of gastric ischemia) support the adequacy of perfusion and its absence here did not impair outcomes—reinforcing that Doppler remains a gold standard when used judiciously.

Had this collateral pathway been absent or disrupted, gastric ischemia would have likely ensued—necessitating either partial gastrectomy or augmentation via arterial reconstruction or conduit interposition. This underscores the **counterfactual value** of this anatomical variant—not merely as a facilitator but as a critical determinant in preserving organ viability and function.

Our surgical strategy employing CHA clamping prior to pancreatic transection with 12 min of post clamping ischemia time, helped us confirm clinically the gastric serosal colour, capillary refill, and doppler during the phase of clamping allowed real-time assessment of retrograde hepatic and gastric flow. Incorporating intraoperative flow studies, particularly Doppler and ICG where available, may refine our understanding of what constitutes a “resectable” vascular anatomy. This manoeuvre should be considered essential in all DP-CARs, particularly when arterial variants are involved.

The **oncologic outcome** in this patient was encouraging: an R0 resection, two-node positivity, and an uneventful recovery. While literature suggests that DP-CAR is associated with higher morbidity than standard distal pancreatectomy, the potential for improved long-term survival in carefully selected cases justifies the risk [[21], [22], [23]]. This is especially true when anatomical knowledge is leveraged to minimize ischemic complications.

Finally, this case advocates for a future development of vascular perfusion scoring system in the context of DP-CAR, incorporating variant anatomy, arcade integrity, LGA/GDA status, intrahepatic collaterals, and Doppler/ICG data. Such a score could standardize decision-making and predict postoperative perfusion-related complications.

## 4 Conclusion

This case represents a novel demonstration of preserved hepatic and gastric viability after modified Appleby procedure, enabled by an uncommon vascular variant a replaced left hepatic artery originating from a high take-off left gastric artery, both preserved during celiac axis resection. The successful outcome underscores the value of preoperative vascular mapping, intraoperative functional flow validation, and individualized surgical planning.

Our case challenges anatomical dogma by showing that retrograde perfusion from the replaced LHA can maintain adequate gastric and hepatic circulation following celiac axis sacrifice, provided that perfusion is functionally verified. Although long-term radiological follow-up was not feasible, the immediate postoperative course was uneventful and the patient proceeded to adjuvant therapy.

This case exemplifies how arterial intelligence, when combined with technical expertise, can expand the frontiers of resectability in pancreatic cancer and also challenges traditional surgical paradigms by highlighting how vascular anomalies, when understood and leveraged, can transform surgical limitations into opportunities for curative intent.

In Future perfusion-based assessment rather than rigid anatomical exclusion may offer a new paradigm in selecting candidates for extended pancreatectomy, especially in the presence of uncommon arterial configurations.

## What this study adds

**Table:** 

Key domain	Contribution
Anatomical Innovation	First documented instance of retrograde gastric perfusion via a replaced left hepatic artery (LHA) arising from the left gastric artery (LGA) following celiac axis resection.
Surgical Relevance	Demonstrates that meticulous preservation of variant vasculature, combined with intraoperative Doppler validation, can enable extended resectability in pancreatic body cancers previously deemed inoperable.
Technical Insight	Maps a novel collateral pathway: SMA → GDA → PHA → Segment 4 → Segment 2/3 → LGA **via** replaced LHA, preserving perfusion despite gastroepiploic arcade sacrifice.
Clinical Impact	Highlights the feasibility of modified Appleby procedure without ischemic complications, expanding criteria for surgical intervention in locally advanced PDAC.
Educational Value	Underscores the value of 3D CT angiography, intraoperative Doppler assessment, and anatomic intelligence in planning and executing high-stakes HPB resections.

## CRediT authorship contribution statement

• **Dr. Supreet Kumar**
***Primary Surgeon and Lead Author***
  Conceived the case report and performed the surgical procedure. Led preoperative planning, intraoperative decision-making, and postoperative management. Drafted the initial manuscript, integrated literature support, and was responsible for critical revisions and final approval of the version to be published.
• **Dr. Rigved Gupta**
***Co-Surgeon and Surgical Innovation Contributor***
  Assisted in preoperative vascular planning and intraoperative dissection, with a particular focus on anatomical preservation and intraoperative Doppler assessment. Contributed significantly to the interpretation of the unique vascular pathway and reviewed all surgical figures. Participated in manuscript revision, ensuring anatomical accuracy and surgical clarity.
• **Dr. Sandeep Vohra**
***Radiological Lead and Imaging Analyst***
  Performed and interpreted the preoperative contrast-enhanced CT and 3D angiographic reconstructions. Identified the replaced left hepatic artery and mapped the vascular collaterals. Assisted in figure preparation and provided technical narrative for imaging-related sections of the manuscript.
• **Dr. Vivek Tandon**
***Senior Surgical Consultant and Academic Advisor***
  Provided senior oversight for surgical planning and intraoperative strategy. Contributed to postoperative care supervision. Critically reviewed the discussion section and helped align the manuscript with current HPB surgical standards and literature.
• **Dr. Deepak Govil**
***Head of Department and Final Manuscript Guarantor and Lead Surgeon***
  Oversaw all aspects of patient care from diagnosis to discharge. Reviewed the manuscript for oncologic validity, academic integrity, and clinical relevance. Approved the final version and is the guarantor for the authenticity of the report.

## Consent

Written informed consent was obtained from the patient for publication and any accompanying images. A copy is available for review by the Editor-in-Chief on request.

## Ethical approval

Exempted as per institutional protocol for single anonymized case reports.

## Guarantor

Dr Supreet Kumar.

## Research registration number

Not applicable.

## Sources of funding

This research did not receive any specific grant from funding agencies in the public, commercial, or not-for-profit sectors.

## Declaration of competing interest

None declared.
